# Clinician perspectives on how change occurs in multi-family therapy for adolescent anorexia nervosa: a qualitative study

**DOI:** 10.1186/s40337-024-01064-2

**Published:** 2024-07-24

**Authors:** Julian Baudinet, Ivan Eisler, Michelle Roddy, Jasmin Turner, Mima Simic, Ulrike Schmidt

**Affiliations:** 1 Institute of Psychiatry, Psychology and Neuroscience, King’s College London, De Crespigny Park, London, SE5 8AZ UK; 2 Maudsley Centre for Child and Adolescent Eating Disorders, South London and Maudsley NHS Foundation Trust, De Crespigny Park, Denmark Hill, London, SE5 8AZ UK; 3 Adult Eating Disorders Service, South London and Maudsley NHS Foundation Trust, De Crespigny Park, Denmark Hill, London, SE5 8AZ UK

**Keywords:** Anorexia nervosa, Adolescents, Multi-family therapy, Family therapy, Family based treatment, Maudsley family therapy, Qualitative

## Abstract

**Background:**

Multi-family Therapy (MFT) is being increasingly used in specialist eating disorder services internationally. Despite evidence of its efficacy, little is understood about the treatment mechanisms and what specifically promotes change. This study aimed to understand clinician perspectives on how change occurs during MFT.

**Methods:**

Clinicians with (a) 5 or more years’ experience facilitating MFT and (b) who had facilitated a minimum of two MFT groups were eligible for this study. Two individual interviews and four semi-structured focus groups were conducted online. Recordings were transcribed verbatim and analysed using reflexive thematic analysis.

**Results:**

Twelve clinicians (five systemic/family psychotherapists, five clinical psychologists, and two consultant child and adolescent psychiatrists) from six different specialist services in the UK participated. Four main inter-connected themes describing how change is perceived to occur were generated; (1) Intensity and immediacy, (2) Flexibility, (3) New ideas and channels of learning and (4) Containment.

**Conclusions:**

Current data matches closely with young person and parent experiences of MFT and intensive day treatment and how they perceive change to occur. Quantitative data are now needed to evaluate the impact of these factors on outcome.

**Plain English Summary**

Multi-family Therapy (MFT) is being increasingly used in specialist eating disorder services internationally. While there is evidence that it is helpful, little is understood about how the treatment works and what specifically promotes change. This study aimed to understand how clinician believe change to occur during MFT for young people and their family members. Clinicians with (a) five or more years’ experience facilitating MFT and (b) who had facilitated a minimum of two MFT groups were eligible for this study. Two individual interviews and four semi-structured focus groups were conducted online. Recordings were written out word-for-word and analysed using reflexive thematic analysis, a commonly used method for analysing this type of data. Twelve clinicians (five systemic/family psychotherapists, five clinical psychologists, and two consultant child and adolescent psychiatrists) from six different specialist services in the UK participated. Four related themes describing how change is perceived to occur were generated; (1) Intensity and immediacy, (2) Flexibility, (3) New ideas and channels of learning and (4) Containment. Current data matches closely with young person and parent experiences of MFT and intensive day treatment and how they perceive change to occur. These factors now need to be tested in future research.

## Background

Multi-family therapy (MFT) is a group-based intervention that is becoming increasingly widespread internationally [36]. It involves several families, typically between four and nine, joining together for treatment with the support of two or more multi-disciplinary professionals. Early developments in MFT for eating disorders originate in Dresden, Germany [1, 43] and London, UK [21]. While models vary internationally, MFT is often more intensive than typical outpatient treatment and other brief intensive treatment programs [27, 35, 41], but less intensive than day programmes/partial hospitalisation programmes [10] and residential treatments [30, 40]. In the outpatient context, MFT usually includes several hours of clinical contact over several consecutive days [3].

MFT has been part of adolescent eating disorder treatment since the 1990s [3, 4, 53]. Different versions have been described for adolescent (age 10–18 years) anorexia nervosa (MFT-AN) [11, 44] and bulimia nervosa (MFT-BN) [26, 47]. MFT is also recommended by some international guidelines in the treatment of adolescent anorexia nervosa [20, 34, 39].

Empirical data exploring the outcomes and experience of MFT are beginning to emerge. Several case series in both research and community settings [32, 38, 46, 48, 52], as well as one outpatient randomised controlled trial (RCT) (N = 169; [24]) have been published. Two further RCT protocols have also published [4, 8, 11, 18], suggesting more is expected although data are yet to be reported. As an adjunct to family therapy, the typical first-line recommended treatment for adolescents with eating disorders, MFT is associated with improved outcomes [24]. In their 12-month RCT comparing single family therapy alone with (experimental arm) or without (control arm) the addition of up to 10 days of MFT, participants who received MFT-AN were significantly more likely to have a better global outcome at the end of treatment, using the Morgan Russell outcome criteria [42]. At 6-month follow-up (18 months post-randomisation) this difference was no longer significant, however, weight was significantly higher for those who received MFT-AN compared to single-family therapy [24]. In a post-hoc moderator analysis of these data, families presenting to treatment with reduced positive caregiving experiences had improved outcomes in MFT compared to single family therapy [9].

Despite this emerging empirical evidence base, relatively little is known about the underlying treatment mechanisms. Theoretically, MFT is rooted in eating disorder focussed family therapy [14, 23, 44], which hypothesises that recovery occurs in the context of (1) a secure, trusting relationship with your therapist and team, (2) the active involvement of parents in supporting their child to eat and manage distress early in treatment, and (3) supporting the young person to individuate in a developmentally appropriate way in the later parts of treatment. Nevertheless, the MFT theoretical model has had little empirical attention.

The addition of MFT to family therapy has been hypothesised to build upon these factors by reducing isolation, providing new opportunities for learning in a peer-to-peer context and by increasing treatment intensity, early in the process [22, 44]. Qualitative data from the young person and parent/caregiver perspective suggest MFT is perceived as unique, challenging and helpful, with the group context and intensity key to promoting change [5, 7, 9, 12, 25]. Two publications, which appear to use the same sample (n = 6 clinicians), have explored the clinician perspective to date. They focus on the implementation of MFT in services and systemic change associated with treatment [50, 51]. While informative, the clinician and parent/caregiver data were reported together, making it difficult to understand the unique perspectives of each.

This study aims to add to this emerging evidence base by exploring how experienced MFT clinicians perceive change to occur for the young people and families during MFT-AN. This will complement data on the perceived change processes within MFT-AN reported from the young person and parent perspective [5, 7, 9] and aid future research design, as very little is known about the MFT treatment mechanisms.

## Method

Ethics approval was granted for this project by the Stanmore Research Ethics Committee London (IRAS: 234354; REC: 20/LO/0839). All participants provided informed consent to participate and publication of the findings, including the use of anonymised quotes.

### Sample

Mental health clinicians who had (a) five or more years’ experience facilitating MFT groups and (b) facilitated a minimum of two MFT groups were eligible for this study.

### Recruitment

Potential participants were identified from three sources:

1. Current staff at the Maudsley Centre for Child and Adolescent Eating Disorders (MCCAED) a specialist eating disorder service who were part of developing the treatment and provides regular training and supervision in MFT.
2. Clinicians who worked on the most recent MFT trial conducted in the UK [24].
3. Clinicians who have previously attended MFT training delivered by MCCAED.

Clinicians from each of these three sources were approached via email. Those interested were provided the information and consent forms and an opportunity to discuss the study with one of the researchers.

### Data collection

All participants provided informed consent. Four semi-structured qualitative focus group interviews were conducted between July 2022 and February 2023 using online video-conferencing technology (Microsoft Teams). Recordings were then transcribed verbatim. All were conducted by authors MR (cisgendered female, white Australian, assistant psychologist, BPsychSci, PGDip, MSocSc) and JT (cisgendered female, white British, assistant psychologist, BA(hons.)). Each interview lasted approximately 90 min with only the two interviewers and participants present (Pilot 1, 1 participant; Pilot 2, 1 Participant; Group 1, 3 participants; Group 2, 3 participants; Group 3, 2 participants; Group 4, 2 participants). MR and JT had some theoretical knowledge of MFT and had attended theoretical and experiential training in MFT. MR also had one week of clinical experience as a helper in MFT at MCCAED. JT did not have any clinical experience of the treatment. Focus groups were recorded and then transcribed verbatim. These were not returned to participants for review.

To ensure uniformity, an interview topic guide was given to both interviewers and two pilot interviews with individuals (i.e. not groups) were conducted prior to data collection. No amendments were made to the interview schedule based off the experience from offering the pilot interviews. JT had previous experience facilitating qualitative interviews. MR did not. Pilot interviews were used as an opportunity to practice facilitation and to reflect with the research team afterwards about ensuring uniformity whist also exploring participants’ responses.

### Analysis plan

Data generated were analysed by authors JB (cisgendered male, white Australian, clinical psychologist, DClinPsy/PhD) and JT using reflexive thematic analysis [16]. A critical realist framework was adopted when analysing the data. Experiences were considered subjective and inextricably influenced by cultural and social context. JB approached the data with extensive knowledge of MFT and many years of experience clinically delivering, writing about and teaching on the MFT model. Conversely, JT approached the data with no clinical experience, but some theoretical knowledge, of the treatment. These differences in analysers’ level of experience with MFT was considered a strength of the analysis and provided new insights and depth to the findings.

JB and JT followed the six phases of reflexive thematic analysis [15, 16]. They initially familiarised themselves with the data (step 1) by reading and re-reading all transcripts. Thereafter, each independently coded (step 2) and then generated initial themes (step 3) based on wider possible patterns of meaning. The analysing authors met over two separate one-hour meetings and email correspondence to discuss these. Via an iterative process revised preliminary themes (step 4) were then reached, followed by a final consensus on themes (step 5) and writing of the manuscript (step 6).

The approach to the data was deductive and flexible [28], drawing on existing MFT theory and literature. For example, it was expected that participants would talk about the unique group-based format of MFT and its impact on change. Nevertheless, these expectations did not restrict the approach to coding. The concept of data saturation was not used during the analysis as it was recognised that different meanings are generated by different researchers and the process is inescapably subjective [17]. No sample size calculation was conducted due the lack of guidance and consensus on how to identify an appropriate sample size for thematic analysis [31]. Available guidance indicates decisions around sample size are influenced by the study aims, sample specificity, use of theory, quality of dialogue and type of analysis [37]. Given the specific nature of the research question, the participant mix of profession, their place of employment, and use of theory, twelve was deemed appropriate for this study. No software was used to assist analysis.

### Member checking

Synthesized member checking was completed in line with suggested guidance [13]. After the analysing authors generated themes from the data, all participants were sent a summary and were given an opportunity to respond via email about whether the results reflected their experiences and whether there was anything they would amend or add. 10/12 (83.3%) participants responded. All confirmed that the themes fitted well with their experience and understanding of change within MFT and did not suggest any modifications to the themes or figure generated.

## Results

A total of 62 potentially eligible clinicians were approached (10 from MCCAED, 2 of which were from the Eisler et al. [24] trial, and 52 from the MFT-AN training attendee list). Of the 62 contacted clinicians, 12 (19%) consented and participated. Of the remaining 50 clinicians, 16/50 (32%) actively declined, 3/50 (6%) passively declined (provided initial consent or requested further information but were unreachable or unavailable to complete an interview thereafter), and 31/50 (62%) could not be reached with a maximum of three attempts at contact. Reasons for non-participation included not meeting the minimum criteria to qualify, not having time to participate, not being interested in the study, not feeling confident or experienced enough in the model.

Participants who completed an interview came from six different specialist child and adolescent services in the UK. Five (41.7%) were systemic and family therapists, five (41.67%) were clinical psychologists and two (16.7%) were consultant child and adolescent psychiatrists. None were dually trained (e.g. registered as both a systemic therapist and clinical psychologist). The majority identified as female (10/12, 83.3%) and White (9/12, 75.0%). All identified as cisgendered. None identified as neurodiverse or living with disability. See Table 1 for more details.

**Table 1:** Participant demographics

	N (%)
*Age (years)*	
30–39	2 (16.67%)
40–49	2 (16.67%)
50–59	3 (25.00%)
60–69	2 (16.67%)
Prefer not to say	2 (16.67%)
Missing	1 (8.33%)
*Gender*	
Female	10 (83.33%)
Male	2 (16.67%)
*Ethnicity*	
White	9 (75.00%)
English/Welsh/Scottish/Northern Irish/British	4 (33.33%)
Irish	1 (8.33%)
Any other White background	4 (33.33%)
Indian	1 (8.33%)
Black African	1 (8.33%)
Missing	1 (8.33%)
*Profession*	
Systemic/Family psychotherapist	5 (41.67%)
Clinical psychologist	5 (41.67%)
Consultant child and adolescent psychiatrist	2 (16.67%)
*Place of current employment*	
Specialist child and adolescent eating disorder service	11 (91.67%)
0–25 Specialist Eating Disorder service	1 (8.33%)
*Experience (years delivering)*	*Mean *(*sd*)
Family therapy for anorexia nervosa (FT-AN)	15.69 (9.61)
Multi-family Therapy for anorexia nervosa (MFT-AN)	12.33 (6.16)

*sd* standard deviation

### Qualitative findings

**Fig. 1:**
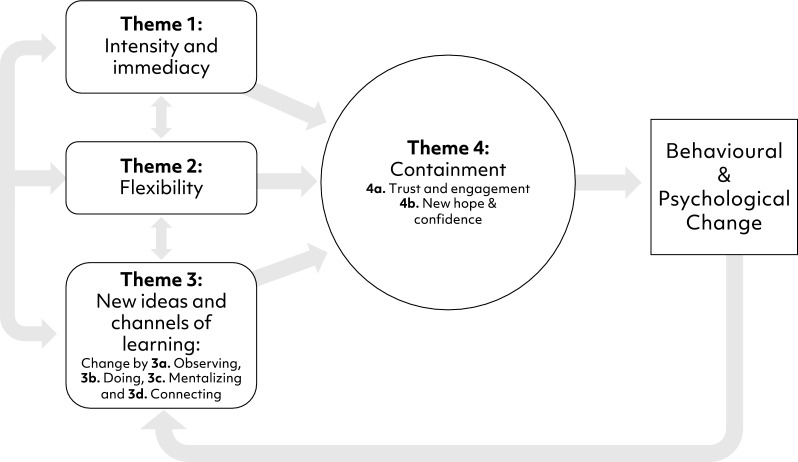
Perceived process of change in multi‐family therapy for anorexia nervosa from the clinician perspective

Four main themes and five subthemes were generated and are understood to be inter-connected. The way these combined to generate change are presented in Fig. 1. The first three themes described perceived processes (1. Intensity, 2. Flexibility and 3. New ideas and channels of learning) that combined to promote the following process (4. Containment). These four factors in combination were then thought to promote behavioural and psychological change.

### Theme 1: intensity and immediacy

There was a sense from all clinicians that bringing families together in a more intensive way was an important element of MFT-AN that helped to create change. People described the combination of people with different but related experiences, the use of activities and the length of time together (four to five full consecutive days) as very ‘powerful’. Combined, these elements helped disrupt illness maintenance factors quickly and engage with each other (within families, with other group members, and the treating team) and in treatment in a new way.

*In terms of mechanism of change, I suppose the diluting [of] the time and [increasing] the intensity has probably got something to do with what makes MFT different. In terms of change, that change can happen faster when you’re in that sort of incubator or, yeah, hothouse situation*

Clinicians spoke of the way the MFT-AN treatment context and intensity directly helped to contain families and created a safe space in which to express emotions and process them in ways that felt hard to do in a the more typical one-hour outpatient family therapy session.

*The other elements of MFT [that] are really key are, sort of, the long days and the intensity … I think it helps break down some habits and some of those patterns that are there that you just can’t do in a one-hour treatment session.*

*The intensity … when you’re together, all these meals every day for four or five days, the things are going to come up. It just sort of feels like it escalates. As long as, you know, you as a therapist can support that, contain that. It’s, you know, I think it’s helpful because it kind of gets things out in the open that maybe might be more easily avoided in sort of weekly sessions … I think we just get to really see … what’s really kind of happening.*

As with anything intense, there are also costs to this. Many clinicians spoke of how this could be exhausting for families and clinicians, alike. Clinicians described a process that families and clinicians move through across the four- or five-day intensive week. While difficult, many thought this was almost needed to promote change. In a way, participants spoke about how MFT is more than the sum of its parts:

*Quite often there’s a little bit of a crisis on the third day [of the MFT intensive week] … on the third day people are feeling really exhausted and like they’re being pushed too hard or it’s all too difficult … And then the change seems to happen on day four or five that … different activities they do all sort of build on one another.*

There was also a sense that if people struggled to engage in the process, they could end the MFT intensive week feeling exhausted and potentially demoralised.

*I think for some people, especially if things are really tricky or that it’s hard to get on the same page, or maybe their relationships are a bit fraught, I think sometimes then it’s harder to feel that hope, and you can leave the week feeling tired and exhausted.*

### Theme 2: flexibility

Clinicians spoke of the importance and benefits of flexibility afforded by MFT, as well as some potential pitfalls. There was a sense that MFT-AN allowed time and space to focus on symptoms (e.g. practical support with meals, tips to manage distress effectively and self-care) and broader issues (e.g. relationships, peer and social difficulties, emotion management) simultaneously and flexibly. One clinician said that: *“in the multi-family context, you get many things happening at the same time, and it’s probably less sequential [than single family therapy]”.* Similarly, there was a sense that the young people and parents can be getting different content and going at different paces as a result of the increased time and ability to break out into smaller groups during MFT. One clinician said MFT-AN allows for: “*different treatment targets for parents and for young people, and they can go into different pace[s].”*

One focus group spoke about valuing the principle-driven nature of MFT-AN and the broader theoretical eating disorder focused family therapy model underpinning it. This group talked about how this helped them to think more clearly with families about what was and maybe was not working so well, and then feeling like they had permission to adapt things based on their formulation.

*Within the model, there is flexibility and scope to be, you know, constantly thinking - are things stuck, why [are] they stuck, is this helpful, is [it] not helpful - and work through it and come through it.*

*I think it’s such a flexible model … The principles remain the principles. But how you work to those principles within the model … if you didn’t like something and you thought it was fundamentally not right, you wouldn’t use it.*

Clinicians described being able to adapt and modify content to best meet the needs of each group. They talked about the paradoxical nature of having the ‘world stop’ to focus on one thing, yet also being able to take time to slowly and deeply process things raised by group members. Relatedly, this also allowed individuals and families to each have different treatment goals and needs met simultaneously during MFT-AN.

For some, this flexibility was also described as generating uncertainty. Not always having a clear roadmap could feel uncontaining at times. The longer treatment days and increased number of people could create unhelpful uncertainty about how best to proceed and which topic to focus on. Several clinicians also described how it could be challenging to know how best to meet everybody’s needs in the group when there were multiple topics raised as important. One clinician said: “*things are dynamic, they are fluid because there’s so much injection of new information from different families.”* Nevertheless, the flexibility and scope to change the schedule to better meet the group’s needs was also seen as a strength:

*The other bit is becoming too manual driven, and I’ve seen [single family] therapists becoming rigid and kind of dead, [which] is mirrored in the family … then the therapist is reinforcing rigidity that already exists in the family anyway because of starvation and rigidity that comes with starvation.*

### Theme 3: new ideas and channels of learning—change by observing, doing and being together

Clinicians also spoke about the way MFT-AN facilitated change by employing new and creative methods of promoting learning, insight and understanding. In addition to talking, clinicians spoke about the way MFT-AN provides the opportunity to learn and make changes through the process of observing and being mirrored in others, trying out new behaviours ‘in-vivo’, and having a more connected, relationally focussed, embodied experience.

*I think that [the] creativity of different techniques is really important. … you’ve got so many people in the family system and everybody learns differently. So if we’re using multiple modalities then actually we’re going to help the learning of everybody … people function differently and … they’ve got different learning styles.*

*It’s creating change, that sort of multiple layer[s] of people doing, seeing, hearing, talking, observing, which happens across the group.*

#### Change by observing

Observing others in MFT-AN was described as helping young people and parents to generate new ideas and understand themselves, their family, and the illness in new ways. As one clinician said: “*simply observing what’s going on for other families is going to be a powerful factor”.* By observing illness-related and recovery-focussed behaviours in others, participants were able to reflect on their current difficulties. Clinicians noticed how hearing ‘old information’ (information already raised in individual family therapy sessions) from a parent or young person in another family could have a much greater impact on people in MFT-AN than hearing the same information from a clinician.

*But sometimes in multi-family therapy, you can recognise it [new solutions] more in other people … it’s learning from the other. It’s easier to see it in other people than it is to see it in ourselves, isn’t it?*

Clinicians spoke about how participants might observe others in the group and either recognise something they were doing or see something they wanted not to repeat.

*That can be a really significant moment. When they [parents] see their child through other people’s eyes and see their child can do things differently when they’re in a different setting with different people.*

#### Change by doing

Similarly, in all interviews and focus groups it was discussed how MFT-AN could provide the unique opportunity to learn by doing. For example, an idea or new way of approaching a difficult situation could be discussed in the morning of an MFT-AN day and then families could try it out in an upcoming meal or break time. This was particularly noted around mealtimes and supporting families with several meals per MFT day. One clinician remarked that in MFT: *“you’re doing much more around eating. In vivo, here [in MFT] you have multiple occasions [family meals] to help support change.”* The immediacy of this experience was discussed as a key factor that promotes change. By receiving increased, immediate support to try out new behaviours, it was felt that this helped families build motivation and feel more able to continue with managing meals and distress at home.

*[There’s] something about believing, you know, believing because you’re there seeing other families being able to tackle something. … it’s not just the family witnessing … I think it enables people actually to risk more, because they are feeling supported so they can take risks more.*

#### Change by mentalizing

Relatedly, observing others, seeing others in a similar positon and trying out new behaviours was described as helping participants to mentalize more (i.e. able to reflect on ones own mental state and mental states of other people [29]). By becoming more aware of their own and others’ mental states, it was thought MFT helped participants to consider new perspectives and beign to make small changes.

*It feels that any of the activities that can help take a different perspective are key … Those different opportunities throughout the week to sort of keep having, sort of, trying to see things from a different perspective, and I think the cumulative effect of doing those multiple times over a week … and seeing other people, you know, you might not be in the role play in the middle of the circle, when you’re doing it, but, you know, the other families are also witnessing that role reversal, and it’s getting them thinking.*

#### Change through connection

In addition to new learning through observing and doing, clinicians in this study also spoke about how important the embodied, connected nature of MFT-AN was in creating change. The activity-based nature of MFT-AN was described as helping young people and families not just ‘*hear’* what was needed but *‘know’* (or embody) it. One participant described the how: *“there’s sort of this idea of, sort of hope, and being with other people, there's, there's something powerful about that, I think that can mobilise people.”*

This process was partly enacted through the use of non-verbal and more action-based activities but was mostly described as being solidified via the new and changing relationships with other group members. Clinicians thought that via this relational focus families felt more contained and, in turn, more able to mentalize, question current patterns of behaviour and being to develop different patterns.

*They [group members] feel understood by them [each other], and so they will hear what other people would say more clearly, but will also challenge things that otherwise would be ignored.*

### Theme 4: containment

The aforementioned three themes were described by participants as combining to contribute towards containment and engagement of individuals, families and the group as a whole. Clinicians described how a sense of a) trust and engagement and b) new hope and confidence followed.

*[The] multi-family therapy context adds another way of containing what’s happening, a sort of … the containment of the anxiety is not just something that’s brought in by the therapist and the multi-disciplinary team, but it’s also something that the group as a whole engenders.*

#### Trust and engagement

There was a strong sense in all interviews that MFT helped young people and families to trust the multidisciplinary team more, as well as each other and themselves. This was not immediate, and could take several days to develop, however, once it occurred, it was thought to be a very powerful and essential factor for promoting change.

*I feel that there is a moment where the group develops a trust and it’s in the first days, it’s not in the first day and it’s not in the second day. It’s often on the third day, … there’s a sort of, it feels like you can feel it in the room when it comes to a point where the families have trust in each other and in the therapy team, and it’s going somewhere.*

Participants discussed how MFT could function as a ‘safe base’. This was thought to be the case for all members of the group, clinicians included. It was from this base that experimentation and new changes could be made towards recovery.

*MFT has a role of providing a safe base, not only for the family but for the therapist. The therapist has to feel like they have a team somewhere who’s going to support them, talk things through and have their back, cheerlead.*

Clinicians spoke about how families say to them that talking to the graduate family makes them think: *together we can maybe trust in the process more because it’s not just us saying it works. It’s someone who’s actually been through it, has found it helpful and challenging.*

#### New hope and confidence

A key process was also the ability for MFT to instil hope. Via the processes described above, clinicians spoke about how they combined to create confidence and new hope in the possibility of recovery and a future without the illness. This was thought to start at the beginning of MFT at the introductory afternoon, specifically when families about to start MFT meet the graduate family.

*It [MFT] just instils that sense of possibility, doesn’t it? When they see that graduate family [the families] just imagined it to be possible - to recover - just to see a family, togetherness, the way they were able to talk, the kind of wellness. …*

This was described as then continuing throughout the MFT week and beyond into treatment. In the same way that trust and engagement developed, so did confidence and new hope, in parallel:

*I think there’s sort of this journey where people come into the 4 or 5 day [MFT] intensive where they come in on their best behaviour. By the middle of the week, they’re quite distressed. We see some sort of a lull in energy and feeling like the task is really big. And then by the end of the week, I think sort of hope can pick up again and the spirit can, can increase. And I think, I don’t know if it’s about any specific activities, but it’s more about working through that process over the weeks. I think by the end of the 4 days, often people leave with a real sort of spring in their step, feel like they’ve got some new ideas and some skills and they are able to work on something and maybe they’ve had a bit of success or they’ve tried a few things out, even though there’s obviously probably been really hard things during that week as well.*

This was something considered quite specific to MFT and difficult to attain in single-family therapy. Clinicians attributed this to a combination of all the elements discussed and kept coming back to the idea that the whole is greater than the sum of its parts.

*Hope is easier to instil and get back in MFT than in single family therapy. I think that’s maybe the difference in mechanism of change because you usually finish the first, whatever, four or five days in MFT … [and] there’s usually hope by the end of MFT, and it’s palpable.*

## Discussion

The aim of this study was to explore clinician perspectives on how change occurs for young people and family members during MFT. Four themes were generated that mapped onto perceived change factors. All were inter-related and influenced each other. The first three (1. Intensity, 2. Flexibility and 3. New channels of learning) were described as combining to generate the fourth (4. Containment). From this platform of containment, psychological and behavioural change was thought to occur. These process also seem to complement and possibly activate the perceived change processes within single family therapy for anorexia nervosa (cf [6]).

The current findings match closely with MFT theory, which emphasises the unique ‘hot house’ environment, embodied nature of the treatment and addition of peer support as all contributing towards change [2, 7, 43–45]. It also fits with recent findings that families presenting to treatment with lower parent-rated positive caregiving experiences have better outcome in MFT, compared to single-family therapy alone. These data suggest the group-based MFT context may help support parents who present with greater caregiving burden.

It also maps closely, although not exactly, onto recent qualitative data on the perceived change processes from the perspective of young people and parents/caregivers who have received MFT-AN [5, 7, 9] and combined clinician/parent data [50, 51]. All three groups (young people, parents and clinicians) describe the importance of intensity, connection with others and the new learning afforded during MFT. Slight differences were noted, however, in the emphasis placed on ‘*comparisons’* that the young people and parents spoke about. While similar to the clinician subtheme of ‘*change by observing’*, young people and parents specifically spoke about not just noticing and considering others in the group, but actively comparing themselves to others in both positive and negative ways [5, 7, 9]. Certainly, families and young people spoke about the importance of feeling supported. The importance of containment also seems to link closely with the concepts of epistemic trust (how much one trusts and is willing to consider information provided by others [29]) and relational containment within families [49], both of which have been hypothesised as important for change to occur in single family therapy for anorexia nervosa.

Additionally, clinicians spoke much more about the concept of flexibility, containment and safety than the young people or parents. Flexibility did not feature much for young people and parents and containment was not mentioned. This may be, in part, due to psychological theory and language influencing the way clinicians describe the experience, as opposed to the concept not being relevant. Alternatively, without anything to compare to, perhaps it is harder for young people and parents/caregivers to be aware that the treatment they received is more or less flexible than other treatments.

The current findings also map on relatively closely onto recent qualitative data on young person and parent experiences of intensive day programme treatment [19, 33]. While considerably different treatments with different durations, the similarities in how patients perceive both intensive treatments to enable change is striking. Most similar was the importance of connecting with others who have had similar experiences, the importance of receiving both practical as well as emotional support, the benefits of trying out new behaviours with support and then practicing them more broadly, and the powerful impact of feeling supported by a team of responsive and knowledgeable clinicians.

Given the similarities, it gives greater weight to the findings that there is something important about offering intensive support that includes peer support with a combined practical and emotional focus. Empirical investigation of these themes is now needed to try and quantify their impact, to determine which may be the most essential elements of treatment, whether they universally apply to all, and whether they occur in parallel, combination or sequentially. Another area of future research will be to understand perceived change processes in less intensive treatments (e.g. individual or single-family therapies) that do not have these additional elements of intensity and peer support.

### Strengths and limitations

A strength of this study is the breadth of services that the clinicians worked in. While the sample size was relatively small, participants spanned six specialist child and adolescent services, which helps to ensure a diversity of perspectives. Nevertheless, the participants were mostly white and all psychologists, family therapists or psychiatrists. It would be good to gather further data from clinicians with different training and professional backgrounds (e.g. nursing). Furthermore, it would also be helpful to gather data from clinicians with greater personal diversity given that participants and the authors that ran the focus groups were predominantly white females. Another strength is that the current study extends findings from a similar study conducted with young people and parents [5, 7, 9]. Having multiple perspectives on how change might occur adds richness to the literature and brings nuance to our understanding of MFT treatment processes. A figure combining these perspectives are presented in a recent MFT review article [3]. A significant limitation is that these data are not linked to quantitative data or outcomes. This requires further investigation. Additionally, given the similarity of factors generated in the current study to other intensive treatments, it will be important to better understand whether some are unique to MFT only, or whether multiple forms of intensive treatment lead to similar outcomes. Lastly, many participants approached did not participate or could not be reached, introducing potential bias in the sample.

## Conclusion

The current study adds to the existing literature on MFT for adolescent eating disorders. It confirms and extends previous data gathered from young people and parents who have received MFT by suggesting intensity, flexibility, additional modes of learning and containment are all key factors that promote change within the treatment.

## Data Availability

Data will be made available upon reasonable request for those participants who have consented to this.
